# Chiral and degenerate perfect absorption on exceptional surfaces

**DOI:** 10.1038/s41467-022-27990-w

**Published:** 2022-02-01

**Authors:** S. Soleymani, Q. Zhong, M. Mokim, S. Rotter, R. El-Ganainy, Ş. K. Özdemir

**Affiliations:** 1 Department of Engineering Science and Mechanics, The Pennsylvania State University, University Park, PA 16802 USA; 2 Department of Physics and Henes Center for Quantum Phenomena, Michigan Technological University, Houghton, MI 49931 USA; 3 Institute for Theoretical Physics, Vienna University of Technology (TU Wien), A–1040 Vienna, Austria; 4 Materials Research Institute (MRI), The Pennsylvania State University, University Park, PA 16802 USA

**Keywords:** Micro-optics, Nanophotonics and plasmonics, Quantum optics

## Abstract

Engineering light-matter interactions using non-Hermiticity, particularly through spectral degeneracies known as exceptional points (EPs), is an emerging field with potential applications in areas such as cavity quantum electrodynamics, spectral filtering, sensing, and thermal imaging. However, tuning and stabilizing a system to a discrete EP in parameter space is a challenging task. Here, we circumvent this challenge by operating a waveguide-coupled resonator on a surface of EPs, known as an exceptional surface (ES). We achieve this by terminating only one end of the waveguide with a tuneable symmetric reflector to induce a nonreciprocal coupling between the frequency-degenerate clockwise and counterclockwise resonator modes. By operating the system at critical coupling on the ES, we demonstrate chiral and degenerate perfect absorption with squared-Lorentzian lineshape. We expect our approach to be useful for studying quantum processes at EPs and to serve as a bridge between non-Hermitian physics and other fields that rely on radiation engineering.

## Abstract (web summary)

Robust engineering of non-Hermitian light-matter coupling will be crucial for future optical device design. Here the authors present a photonic system that operates on an exceptional surface, demonstrating chiral and degenerate absorption with super-Lorentzian lineshape.

## Introduction

Exceptional points (EPs) are generic degeneracies of non-Hermitian systems, where two or more eigenvalues and the associated eigenvectors of a system coalesce, resulting in the reduction of the system’s dimensionality and in a severely skewed vector space^1–6^. This is very different from Hermitian degeneracies known as diabolic points where eigenvectors stay orthogonal to each other although the eigenvalues are degenerate^1–8^. This difference has created a variety of novel opportunities and attracted enormous attention from different scientific disciplines. Among the intriguing phenomena at or in the vicinity of EPs are chiral behavior^9^, enhanced response to small perturbations^10–17^, and enhanced transmission and lasing with increasing loss^18–20^, just to name a few [see references^2–6^ for a complete list].

EPs emerge in systems through different routes, such as balanced loss and gain as in parity-time (PT) symmetric systems^21–24^, judiciously engineered loss imbalance in loss-only systems^18,19,25,26^, asymmetric coupling between modes of a system (e.g., clockwise CW and counter-clockwise CCW modes)^9,10^, post-selection in quantum systems^27,28^, and parametric modulation^29^, all of which have been demonstrated in experiments. The latter three routes differ from the former two routes because they do not rely on introducing additional loss or gain into the system, and thus remain free from the associated noise contributions. As such they have the potential to be used in sensors^11,30,31^, spontaneous emission control^32,33^, and other fields where noise imposes stringent constraints.

**Fig. 1:**
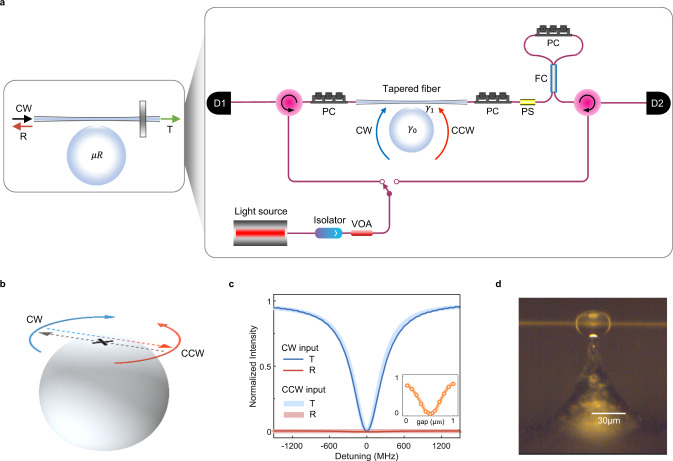
Experimental setup for tunable unidirectional coupling between CW and CCW modes of a microresonator. **a** A partially reflecting mirror placed at only one end of a waveguide (end-mirror) in a waveguide-coupled microresonator ($\mu R$) system unidirectionally couples the frequency-degenerate clockwise (CW) and counter-clockwise (CCW) modes of the resonator. Such an end-mirror with tunable reflection coefficient is implemented as a fiber-loop mirror using a fiber coupler (FC) and polarization controller (PC). ${\gamma }_{1}$: waveguide-resonator coupling loss; ${\gamma }_{0}$: sum of all resonator-related losses such as radiation, scattering, and material losses; D1 & D2: photodetector; VOA: variable optical attenuator. **b** In the setup in (**a**), the CW mode couples to the CCW mode, but the CCW mode does not couple to the CW mode. This unidirectional (nonreciprocal) coupling between the CW and the CCW modes of the resonator creates an exceptional surface (ES). Controlling the phase and the magnitude of the reflected light from the end-mirror allows steering the system on the ES. **c** Without the end-mirror, the system has symmetric Lorentzian transmission (blue) and reflection (red) spectra for left and right incidence (CW and CCW inputs). Zero-reflection and the absence of mode splitting in the transmission spectra for both input directions imply the absence of intermodal coupling between the modes. The inset shows that the loading curves are also the same, and the system can be tuned from the overcoupling to the undercoupling regime through the critical coupling point. **d** Optical image of the tapered-fiber coupled on-chip microsphere resonator used in the experiments.

The asymmetric coupling between CW and CCW modes of waveguide-coupled resonators has been achieved in experiments through the control of intermodal scattering by inserting two scatterers in the resonator mode field whose size and relative distance in the field can be tuned^9,26^. This procedure gives rise to isolated EPs that are typically very difficult to stabilize against fabrication errors and fluctuations in the experimental environment. To overcome this problem, the concept of exceptional surfaces (ES) was recently introduced^30,34–36^ and experimentally implemented in magnon polaritons^37^ with potential applications in optical sensing^30^, optical amplification^38^, spontaneous emission control^32^, and optical absorption^39^: these hypersurfaces in parameter space consist of a continuous collection of EPs. An ES emerges in waveguide-coupled ring resonators through unidirectional (i.e., non-reciprocal) coupling between the modes such that the CW mode couples to the CCW but the CCW mode does not couple to the CW mode or vice versa (Fig. 1a, b). This feature provides a stability against unwanted perturbations (e.g., noise, fabrication imperfections, etc.) that typically drive the system away from discrete EPs and thereby deteriorate their most desirable features.

While, in principle, unidirectional coupling in microring resonators can be achieved via back-reflection from a simple end-mirror placed at one of the output ends of the waveguide^28^ (see Fig. 1a), we take a different route here that provides full experimental control over the strength and phase of the back-reflected signal (i.e., we can tune the reflection magnitude and phase of the end-mirror or the reflector), and hence allows us to steer the system on the ES. By critical coupling to the resonator on the ES we observe perfectly absorbing EPs^40^, exhibiting the characteristic quartic absorption lineshape^40^ as the unique hallmark for perfect absorption that is both chiral (i.e., higher absorption for incidence from a specific direction) and degenerate (i.e., two purely incoming wave solutions coalesce at the ES). We emphasize that no additional loss (other than the typical resonator and component losses) is introduced to observe these signatures.

## Results

### Experimental setup and theoretical model

Our experimental system is composed of an on-chip whispering gallery mode microsphere resonator coupled to a tapered-fiber waveguide (Fig. 1d), which is used to couple light in and out of a resonant mode, and a fiber loop with a polarization controller acting as a tuneable end-mirror (Fig. 1a). The resonator supports CW and CCW modes at the same frequency. We have identified a resonant mode with intrinsic quality factor of $7.7\times {10}^{5}$ (measured in the deep undercoupling regime) in the $1440\,{{{{{\rm{nm}}}}}}$ band and confirmed that in the absence of the end-mirror, the transmission (${T}_{{cw}}$ detected at ${D}_{2}$ and ${T}_{{ccw}}$ detected at ${D}_{1}$) and reflection spectra (${R}_{{cw}}$ detected at ${D}_{1}$ and ${R}_{{ccw}}$ detected at ${D}_{2}$) are symmetric for light input in the CW direction (forward or left incidence) and CCW direction (backward or right incidence) (see Fig. 1c): both ${T}_{{cw}}$ and ${T}_{{ccw}}$ exhibit typical Lorentzian lineshapes; ${R}_{{cw}}={R}_{{ccw}}=0$, implying that there is no intermodal coupling between the CW and CCW modes (i.e., no mode splitting); and absorption defined as ${A}_{{cw}({ccw})}=1-{T}_{{cw}\left({ccw}\right)}-{R}_{{cw}\left({ccw}\right)}$ is symmetric (i.e., ${A}_{{cw}}={A}_{{ccw}}$). We have also confirmed that the loading curve is the same for left and right incidence (inputs in the CW and CCW directions) (see Fig. 1c, *inset*). The introduction of the fiber loop acting as a tuneable end-mirror breaks this symmetry by inducing unidirectional coupling between the CW and CCW modes: light transmitted in the forward direction (left incidence; CW mode) is reflected back and couples into the CCW mode, but light transmitted in the opposite direction (right incidence; CCW mode) travels directly to a detector without any back-coupling into the CW mode (i.e., no reflector at the output of the taper in the CCW direction).

Within the context of coupled-mode theory, our experimental setup is described by ${\partial }_{t}A=-i{H}_{{ES}}A$ where $A={({a}_{{cw}},{a}_{{ccw}})}^{T}$, ${a}_{{cw}}$ and ${a}_{{ccw}}$ are the field amplitudes of the CW and CCW modes respectively, and ${H}_{{ES}}$ is the effective Hamiltonian given as

1 $${H}_{{ES}}=\left(\begin{array}{cc}{\omega }_{0}-i\Gamma & 0\\ \kappa & {\omega }_{0}-i\Gamma \end{array}\right).$$

Here, ${\omega }_{0}-i\Gamma$ are the complex frequencies of the degenerate CW and CCW modes, with $\Gamma =\left({\gamma }_{0}+{\gamma }_{1}\right)/2$ corresponding to the cavity loss rate which consists of the waveguide coupling loss ${\gamma }_{1}$ and all other resonator-related losses (i.e., radiation, scattering and material absorption losses) ${\gamma }_{0}$, and $\kappa$ denotes the CW-to-CCW coupling strength. The zero value in the off-diagonal elements implies that the CCW mode does not couple to the CW mode. The unidirectional coupling strength in this system is defined as $\kappa =r{\gamma }_{1}$ with $r=\left|r\right|{{\exp }}(i\phi )$ and $\left|r\right|$ and $\phi$ corresponding to the magnitude and phase of back-reflection from the end-mirror (i.e., fiber-loop mirror). Both the eigenvalues and the corresponding eigenvectors of this system are degenerate and given as ${\omega }_{{{{{\mathrm{1,2}}}}}}={\omega }_{0}-i\Gamma$ and ${a}_{{{{{\mathrm{1,2}}}}}}={(0,1)}^{T}$, forming an EP with CW chirality at the complex frequency ${\omega }_{0}-i\Gamma$. Clearly, the system is at an EP for any non-zero $\kappa$ (i.e., $\left|r\right| \, \ne \, 0$ and ${\gamma }_{1} \ne \, 0$) and for all values of ${\omega }_{0}$ and$\,\Gamma$. Indeed, if $\left|r\right|$ and $\phi$ are steered, the system will trace an ES surface formed by EPs at the complex ES frequency ${\omega }_{0}-i\Gamma$. As such, the system will always stay on a surface even if there are variations both in the amplitude and the phase of $r$. Variations in ${\gamma }_{0}$ and ${\gamma }_{1}$, on the other hand, will create a new ES at a new complex ES frequency differing only in the imaginary part. Similarly, any perturbation (e.g., by temperature) that affects ${\omega }_{0}$ will lead to a new ES frequency differing only in the real part. Thus, although experimental imperfections and fluctuations may shift the complex ES frequency, they will not be able to lift the non-Hermitian degeneracy on the ES and the system will always remain on the surface. We note that any perturbation that will break unidirectional coupling between CW and CCW modes (e.g., a perturbation that changes the zero element in ${H}_{{ES}}$ to a non-zero value $\delta$) will lift the degeneracy, leading to split modes with ${\omega }_{{{{{\mathrm{1,2}}}}}}={\omega }_{0}-i\Gamma \mp \sqrt{\kappa \delta }$, and push the system off the ES.

### Exceptional surfaces and reflection spectra with squared-Lorentzian lineshape

**Fig. 2:**
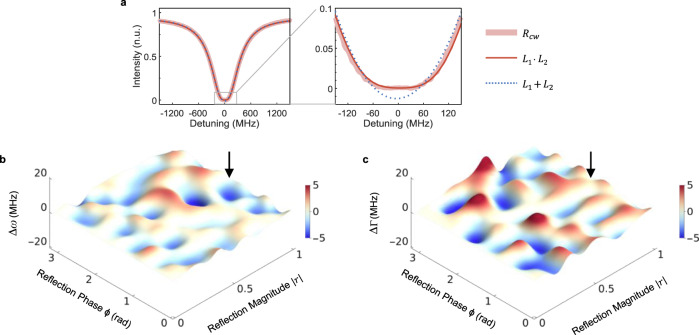
Squared Lorentzian reflection spectra and reconstructed exceptional surface for CW input at critical coupling. **a** Reflection spectrum ${R}_{{cw}}$ obtained for fully reflective end-mirror, $\left|r\right|=1$, has quartic lineshape (left panel). A closer look into the resonance dip of ${R}_{{cw}}$ reveals the flattening of the lineshape (right panel). Best curve fit is obtained using the function $f={L}_{1}\cdot {L}_{2}$ (product of two Lorentzians) rather than $f={L}_{1}+{L}_{2}$ (sum of two Lorentzians). **b**, **c** Reconstructed ES in the 2D parameter space $\left\{\left|r\right|,\phi \right\}$ of the system when the system is operated at the critical coupling for a CW input. Curve fitting to the experimentally obtained ${R}_{{cw}}$ is used to estimate the real and imaginary parts of the complex eigenfrequency of the system at the EP where two eigenfrequencies coalesce. $\triangle \omega$ in (**b**) and $\triangle \Gamma$ in (**c**) corresponds to the difference between the real and imaginary parts of two complex eigenfrequencies obtained from curve fitting. Deviations from zero are attributed to curve fitting noise. Arrows point to the locations of the spectrum on the ES.

We investigated the formation and the stability of an ES in our system by monitoring the reflection and transmission spectra for left and right incidence by tuning the system parameters $\left|r\right|$ (reflection strength), $\phi$ (reflection phase), and ${\gamma }_{1}$ (waveguide-resonator coupling strength) (see Supplementary for theoretical model). We first set the system to critical coupling (${\gamma }_{0}={\gamma }_{1}$), confirmed with zero transmission at the resonance dip both for left and right incidence. Then we vary $\left|r\right|$ and $\phi$, implementing a variable/tunable reflector, and monitor transmission ${T}_{{cw}({ccw})}$ and reflection ${R}_{{cw}({ccw})}$. We observe symmetric transmission spectra ${T}_{{cw}}\equiv {\left|{t}_{{cw}}\right|}^{2}$ and ${T}_{{ccw}}\equiv {\left|{t}_{{ccw}}\right|}^{2}$ with Lorentzian lineshapes for all values of $\left|r\right|$ and $\phi$. These results agree well with coupled-mode theory which predicts ${t}_{{cw}({ccw})}\propto \triangle /(\triangle -i{\gamma }_{0})$ and hence ${T}_{{cw}({ccw})}\propto 1/\left(1+{{\gamma }_{0}}^{2}/{\Delta }^{2}\right)$, where $\Delta$ is the laser-cavity detuning. On the other hand, it is obvious that reflection spectra ${R}_{{cw}}$ and ${R}_{{ccw}}$ will demonstrate an asymmetric behavior because ${R}_{{ccw}}$ is constant at all frequencies (i.e., for right incidence reflection occurs from the mirror only and does not involve the resonator) while ${R}_{{cw}}$ exhibits a resonance. This asymmetric reflection with symmetric transmission for inputs in opposite directions already indicates the presence of an EP. According to coupled-mode theory, the amplitude reflection coefficient for light incident from the left scales as ${r}_{{cw}}\propto {{t}_{{cw}}}^{2}$, leading to ${R}_{{cw}}\equiv {\left|{r}_{{cw}}\right|}^{2}\propto 1/{\left(1+{{\gamma }_{0}}^{2}/{\Delta }^{2}\right)}^{2}$. In other words, the reflection spectrum for left incidence features a squared Lorentzian response. We observe this asymmetry and quartic behavior (i.e., flattening) of the reflection lineshape in our experiments (see Fig. 2a and Supplementary Fig. S6). This type of response indicates the presence of a novel type of perfectly absorbing EP^40^; lineshape modifications associated with such an EP have, however, not been observed up to date because, to the best of our knowledge, all previous experiments have been performed in the vicinity of an EP rather than exactly at an EP due to the difficulty of keeping a system at a discrete EP stably and continuously. Thus, the expected lineshape modification remained obscured and indiscernible in previous works. Our system, on the other hand, operates on an ES and is thus always exactly at an EP even in the presence of experimental imperfections and fluctuations.

To demonstrate that our system is, indeed, on the ES, we have collected ${R}_{{cw}}$ spectra at various values of $\left|r\right|$ and $\phi$ when the system is at critical coupling (${\gamma }_{0}={\gamma }_{1}$) and extracted the frequency and linewidth of the resonance lineshape on the ES. We do this by fitting the experimental data with a function composed of the product of two Lorentzians, that is $f={L}_{1}\cdot{L}_{2}$ with ${L}_{k}={{A}_{k}\triangle }_{k}/({\triangle }_{k}-i{\Gamma }_{k})$, and by estimating $\left\{{\triangle }_{k},{\Gamma }_{k}\right\}$ which should ideally satisfy ${\triangle }_{1}={\triangle }_{2}$ and ${\Gamma }_{1}={\Gamma }_{2}$ on the ES. Plotting the experimentally obtained $\triangle \omega={\triangle }_{1}-{\triangle }_{2}$ and $\triangle \Gamma ={\Gamma }_{1}-{\Gamma }_{2}$ as a function of $\left|r\right|$ and $\phi$ has revealed the ES (see Fig. 2b, c). The $\triangle \omega$ values are in the range $\left[-6.8\,{{\mathrm{MHz}}},3.4\,{{\mathrm{MHz}}}\right]$ and $\triangle \Gamma$ are in the range $\left[-5.4\,{{\mathrm{MHz}}},8.8\,{{\mathrm{MHz}}}\right]$, which, when normalized with the frequency ${\omega }_{0}=207.3\,{{\mathrm{THz}}}$ and the linewidth ${\Gamma }_{0}=502\,{{\mathrm{MHz}}}$ of the resonance at the critical coupling without the end-mirror, yield $\left|\triangle \omega/{\omega }_{0}\right| \, \lesssim \, {10}^{-8}$ and $\triangle \Gamma /{\Gamma }_{0} \, \lesssim \, {10}^{-2}$, implying that the system is, indeed, on an ES.

We have also performed experiments at undercoupling and overcoupling regimes by tuning ${\gamma }_{1}$ (i.e., varying the taper-resonator gap), and observed that the system always stays on an ES and remains robust against changes and unwanted fluctuations in the waveguide-resonator coupling strength (see Supplementary Figs. S7 and S8 for ES formed in the under- and over-coupling regimes). Thus, steering the system in the 2D parameter space using $\left|r\right|$ and $\phi$ always defines an ES regardless of the waveguide-resonator coupling regime. The system will leave the ES only for perturbations that break unidirectionality and establish a symmetric or asymmetric coupling between the CW and CCW modes. We also note that the system is on an ES at all resonances across the spectrum (i.e., one can construct and probe multiple ES in parallel by simultaneously probing multiple resonances).

### Chiral perfect absorption with quartic lineshape on an exceptional surface

**Fig. 3:**
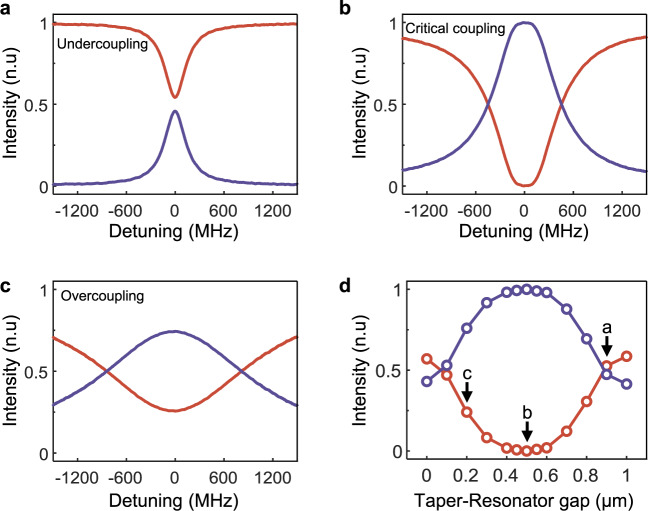
Perfect absorption on an exceptional surface with fully reflective end-mirror. Absorption spectra ${A}_{{cw}}$ (purple curves) are inferred from the measured reflection spectra ${R}_{{cw}}$ (red curves) using ${A}_{{cw}}+{R}_{{cw}}=1$ for a CW input and fully reflective fiber loop ($\left|r\right|=1$). Spectra measured at (**a**), undercoupling regime, (**b**), critical coupling, and (**c**), overcoupling regime reveal perfect absorption on the ES with a quartic absorption lineshape only for critical coupling, as in (**b**). As the system moves away from the critical coupling regime the quartic behavior becomes indiscernible. **d** Measured reflection and calculated absorption exactly at the ES frequency (i.e., zero detuning) for various taper-resonator coupling strength ${\gamma }_{1}$ determined by the gap between the taper and the resonator show the highest absorption at the critical coupling (i.e., gap equals to $\sim 0.5{{\upmu}} {{{{{\rm{m}}}}}}$) where we have ${R}_{{cw}}=0$ and thus ${A}_{{cw}}=1$. As the system moves from critical coupling toward undercoupling or overcoupling regimes, absorption at the resonance monotonously decreases. Circles are the values extracted from experimental data and the straight lines are inserted as guides to the eye. The labelled points in (**d**) are obtained from the spectra shown in (**a**, **b**, and **c**). For a CCW input, all the light is back-reflected by the mirror and no light reaches the resonator, therefore absorption is zero, ${A}_{{ccw}}=0$, and hence chiral absorption on the ES.

Next, we study the absorption properties of the system (see Fig. 1a) operating on the ES for left incidence (input in CW direction) at various taper-resonator coupling conditions when $\left|r\right|=1$ corresponding to a fully reflective end-mirror (see Fig. 3), where ${T}_{{cw}}=0$. Under this condition, the absorption spectrum is calculated using ${A}_{{cw}}=1-{R}_{{cw}}$ where the reflection spectrum is measured by detector ${D}_{1}$. The normalization is carried out by considering the losses ${L}_{{cw}}$, including the insertion and propagation losses when the left incident field travels from the input circulator to the reflector and then back along the same path to ${D}_{1}$ in the absence of the resonator (see Supplementary). The system stays on the ES as we vary the taper-resonator coupling strength, but the absorption strongly depends on the coupling regime, achieving perfect absorption only at the critical coupling (see Fig. 3). This absorption behavior can be explained as follows: Our system operating at the critical coupling with $\left|r\right|=1$ (perfectly reflecting end-mirror) represents a one-channel coherent perfect absorber (CPA) with ${T}_{{cw}}=0$ and ${R}_{{cw}}=1/{\left(1+{{\gamma }_{0}}^{2}/{\Delta }^{2}\right)}^{2}\to 0$ for $\Delta \to 0$, and thus ${A}_{{cw}}=1$ at the ES frequency, thus we refer to this special one-channel CPA as a one-channel CPA-ES. Different from a conventional one-channel CPA (or critical coupling), the resonator is tuned here to an EP on the ES and hence the absorption lineshape is quartic as is the reflection lineshape (see Fig. 3). As the system moves away from the critical coupling point toward the undercoupling or the overcoupling regime, a gradual transition from a squared Lorentzian lineshape to a more Lorentzian-like lineshape is clearly seen (Fig. 3 and Supplementary Fig. S9). As discussed above, our analysis takes the losses into account in the normalization process. If the off-resonance losses ${L}_{{cw}}$ are not accounted for, the absorption will be limited only by ${L}_{{cw}}$, that is ${A}_{{cw}}\to 1-$ ${L}_{{cw}}$ as $\Delta \to 0$, when the system is on the ES at critical coupling.

**Fig. 4:**
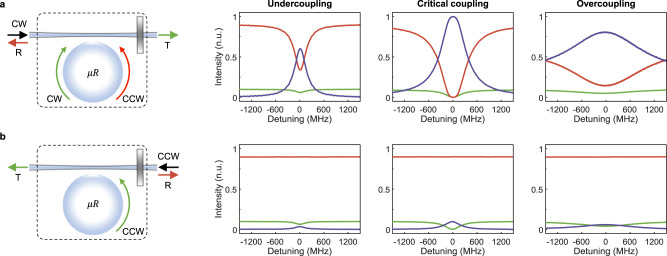
Chiral perfect absorption on an exceptional surface. Dotted boxes in the left panels in (**a** and **b**) represent the ES-device composed of a waveguide-coupled microresonator ($\mu R$) with an end-mirror with 90% reflection and 10% transmission. Black arrows denote the CW and CCW input ports of the ES-device, and red and green arrows represent the corresponding reflection and transmission ports. In the case of CW input as in (**a**), the field inside $\mu R$ has both CW and CCW components whereas it only has a CCW component for the CCW input as in (**b**). Measured transmission ${T}_{{cw}({ccw})}$ (green) and reflection ${R}_{{cw}({ccw})}$ (red) spectra and calculated absorption ${A}_{{cw}({ccw})}=1-{R}_{{cw}\left({ccw}\right)}-{T}_{{cw}({ccw})}$ (purple) spectra of the ES-device at the undercoupling, critical coupling and overcoupling regimes for CW (upper panel) and CCW (lower panel) inputs. ${T}_{{cw}}$ and ${T}_{{ccw}}$ have Lorentzian lineshapes with resonance dips at zero-detuning (ES frequency) at all coupling regimes; ${R}_{{ccw}}$ is constant at all frequencies; and ${R}_{{cw}}$ exhibits squared Lorentzian spectra. Perfect absorption on the ES with quartic lineshape is observed at the critical coupling for CW input only implying chiral perfect absorption. ${A}_{cw}$ is at least ten times larger than ${A}_{{ccw}}$, and hence chiral absorption is realized at all coupling conditions.

We now show that an ES in our system (see Figs. 1a, 4) leads to chiral and perfect absorption also for partially reflecting end-mirrors (see Fig. 1a). The tuneable fiber-loop reflector allows us to construct symmetric mirrors with varying reflection and transmission coefficients. As examples, here we present the results of experiments performed with a 10:90 end-mirror (10% transmission and 90% reflection) and a 50:50 end-mirror (50% transmission and 50% reflection) at different taper-waveguide coupling regimes. The spectra are normalized with the power input to the tapered waveguide for left incidence and with the power just before the reflector for right incidence. We note that varying the reflection phase $\phi$ does not affect the observed features. Typical spectra obtained for the 10:90 end-mirror at different taper-waveguide coupling regimes are shown in Fig. 4 (see Supplementary Fig. S10 for the 50:50 end-mirror).

When the end-mirror is not 100% reflecting, we have access to reflection and transmission spectra $({T}_{{cw}},{R}_{{cw}})$ and $({T}_{{ccw}},{R}_{{ccw}})$ for left (CW direction) and right incidence (CCW direction) from which the normalized absorption spectra ${A}_{{cw}}$ and ${A}_{{ccw}}$ can be calculated using the expression ${A}_{{cw}({ccw})}+{R}_{{cw}({ccw})}+{T}_{{cw}({ccw})}=1$. In the experiments, ${T}_{{cw}({ccw})}$ for left and right incidence exhibit typical resonance dips at the ES frequency with Lorentzian lineshapes. However, reflection spectra differ significantly: ${R}_{{cw}}$ has a squared Lorentzian lineshape with a flattened resonance dip around the ES frequency (see Fig. 4) whereas ${R}_{{ccw}}$ is constant ($e.g.,{R}_{{ccw}}=0.9$ for the 10:90 end-mirror and 0.5 for the 50:50 end-mirror) at all frequencies because it does not involve the resonator (see Fig. 4). The chirality in this behavior (asymmetry in reflection) stems from the larger absorption for the left incidence compared to the right incidence and the degeneracy in the absorption is the result of the strong coupling between the CW and CCW modes of the resonator for left incidence (no coupling between them for right incidence). Indeed, the absorption spectrum ${A}_{{cw}}$ for CW input (left incidence) is a superposition of a Lorentzian term coming from the transmission ${T}_{{cw}}$ and a squared-Lorentzian term from ${R}_{{cw}}$, whereas ${A}_{{ccw}}$ for CCW input is always a Lorentzian (see Supplementary). The weights of the Lorentzian and squared-Lorentzian terms in the superposition are determined by the reflectivity of the end-mirror (i.e., ${A}_{{cw}}$ is squared Lorentzian for a 100% reflecting mirror and it is Lorentzian for a 0% reflecting mirror) and hence the effective unidirectional coupling $\kappa$ between the CW and CCW modes. Another parameter that affects $\kappa$ and thereby the contribution of Lorentzian and squared-Lorentzian terms to the final lineshape is the waveguide-resonator coupling strength ${\gamma }_{1}$ through the expression $\kappa =r{\gamma }_{1}$. Thus, when the taper-resonator coupling or the reflectivity of the end-mirror is varied, the system continues to be on an ES but the lineshape and the overall amount of the absorption are altered.

Interestingly, a gradual transition from a quartic (squared-Lorentzian) form to a quadratic (Lorentzian) form in the ${A}_{{cw}}$ lineshape takes place as the taper-resonator coupling moves from the critical coupling toward the undercoupling or overcoupling regime (see Fig. 4, upper panel) or the reflectivity of the end-mirror is tuned. Perfect absorption with flat-top squared Lorentzian lineshape is clearly seen when the system is at the critical coupling and the input is CW (see Fig. 4).

### Tuning chiral absorption and its bandwidth on an exceptional surface

**Fig. 5:**
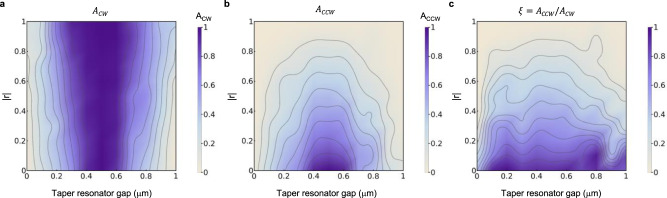
Tunable chiral absorption on an exceptional surface by varying reflection from the end-mirror or the waveguide-resonator coupling strength. **a**, **b** The amount of absorption at the ES frequency for left (CW input) and right (CCW input) incidence can be tuned by controlling the taper-resonator gap and the reflectivity $\left|r\right|$ of the end-mirror, implemented with the fiber loop with a polarization controller. **c** Absorption ratio ${\xi =A}_{{ccw}}/{A}_{{cw}}$ of right incidence to left incidence at the ES frequency. CPA-ES is obtained at the critical coupling for left incidence. Critical coupling is achieved when the taper-resonator gap is $\sim {\!}0.5\,{\upmu} {{{{{\rm{m}}}}}}$, with the gap smaller than $0.5\,{\upmu} {{{{{\rm{m}}}}}}$ corresponding to overcoupling and a gap larger than 0.$5\,{\upmu} {{{{{\rm{m}}}}}}$ corresponding to undercoupling.

Finally, we steer the system on the ES and determine the absorption ${A}_{{cw}}$ and ${A}_{{ccw}}$ at the ES frequency for left (see Fig. 5a) and right (see Fig. 5b) incidence, respectively, by tuning the fiber-loop mirror parameters and the taper-resonator coupling. We present the results in Fig. 5 demonstrating that perfect absorption on an ES (${A}_{{cw}}=1$ at resonance with squared-Lorentzian lineshape) takes place for left incidence at the critical coupling for all reflectivity values $\left|r\right|$ of the end-mirror (Fig. 5a). For right incidence, on the other hand, conventional perfect absorption (${A}_{{ccw}}=1$ at resonance with Lorentzian lineshape) occurs at the critical coupling only for $\left|r\right|=0$ (i.e., completely transmitting end-mirror), and ${A}_{{ccw}}$ decreases with increasing $\left|r\right|$. As the taper-resonator gap increases from zero (i.e., overcoupling), ${A}_{{cw}}$ and ${A}_{{ccw}}$ first increase reaching their maximum value at the critical coupling, and then start decreasing as the gap increases (the system moves toward deep undercoupling regime). Chirality of absorption can be better seen in the ratio $\xi ={A}_{{ccw}}/{A}_{{cw}}$ (Fig. 5c) which can be tuned in the range $\left[{{{{\mathrm{0,1}}}}}\right]$: $\xi =0$ denotes no absorption for right incidence (${A}_{{ccw}}=0$) corresponding to a fully reflecting end-mirror; $\xi =1$ denotes equal absorption for incidence in both directions (${A}_{{ccw}}={A}_{{cw}}$); and all other values of $0 \, < \, \xi \, < \, 1$ imply chiral absorption, that is ${A}_{{cw}} > {A}_{{ccw}}$, with higher chirality at higher $\left|r\right|$ and at taper-resonator gaps closer to the critical coupling condition.

It is worth noting that ES does not only enable chiral absorption at the ES frequency, but the involved degeneracy also provides a way to control the absorption bandwidth. We have observed that the absorption bandwidth defined as full-width at half-maximum is different for left and right incidence (i.e., chirality in bandwidth). More interestingly, we have found that compared to the conventional one-channel CPA at critical coupling, the absorption bandwidth on the ES is $1.26$, $1.41$, and $1.59$ times larger for 50:50, 10:90, and fully reflecting end-mirrors, respectively. These values are close to the theoretically predicted values of $1.27$, $1.50$, and $1.55$. The flattened absorption spectrum in the vicinity of critical coupling for left incidence may provide a remedy to the narrow absorption bandwidth of a conventional one-channel CPA (i.e., a waveguide-coupled resonator operating at critical coupling)—a problem that has hindered progress in technologies relying on CPA.

## Discussion

In this work, we have demonstrated a non-Hermitian optical device which exhibits an ES and chiral perfect absorption. Since the device operates always exactly at an EP when on the ES, it provides a stable and controllable platform to study EP-related phenomena and processes, revealing chiral and degenerate perfect absorption on an ES with quartic reflection and absorption lineshapes as the defining hallmark. Our results will pave the way toward control of various optical processes and light-matter interactions exactly at an EP (not limited to the spectra in the vicinity of an EP), with potential applications ranging from chiral light-matter interaction, lasing, and emission to chiral nonlinear photonics and photovoltaics. Creating the ES through a simple unidirectional coupling route between two modes can be extended to other physical platforms where the coupling between modes and systems can be made unidirectional by electrical, optical, photonics or acoustic feedback or back-reflection. Since no additional loss or gain is introduced into the system, the ES obtained through unidirectional coupling can also benefit studies of quantum dynamics in non-Hermitian systems.

## Supplementary information

Supplementary Material

## Data Availability

The datasets that support the finding of this study are available from the corresponding author upon reasonable request.
