# Supplementary material for: Chiral and degenerate perfect absorption on exceptional surfaces
Source: Nat Commun. 2022 Feb 1;13:599. doi: 10.1038/s41467-022-27990-w (PMC8807609; doi:10.1038/s41467-022-27990-w)
Supplement: Supplementary file 1 — Supplementary Material [file 41467_2022_27990_MOESM1_ESM.pdf]

Supplementary Materials

for

Chiral and Degenerate Perfect Absorption on Exceptional Surfaces

S. Soleymani,[1] Q. Zhong,[2] M. Mokim,[1] S. Rotter,[3] R. El-Ganainy,[2] & Ş. K. Özdemir,[1,4]

[1] *Department of Engineering Science and Mechanics, The Pennsylvania State University, University Park, PA 16802, USA*

[2] *Department of Physics and Henes Center for Quantum Phenomena, Michigan Technological University, Houghton, Michigan, 49931, USA*

[3] *Institute for Theoretical Physics, Vienna University of Technology (TU Wien), A–1040 Vienna, Austria*

[4] *Materials Research Institute (MRI), The Pennsylvania State University, University Park, PA 16802, USA*

I. Experimental setup and theoretical model

Here we introduce the experimental and the theoretical model in detail and provide information on the processing of the experimentally collected data. We derive the expressions for transmission, reflection, and absorption spectra of our experimental system for the inputs in the clockwise (CW) and counterclockwise (CCW) directions and provide simulation results to clarify the effects of various experimental parameters.

a) *Experimental setup*

Schematics of the setup is given in **Fig. S1**. Light from a tunable laser (1440nm band) is used to probe the transmission and reflection spectra of a waveguide-coupled resonator. Our resonator is an on-chip whispering-gallery-mode (WGM) microsphere resonator, which is fabricated through CO_2 laser reflow of a silica microdisk resonator. The waveguide used to couple light in and out of the resonator is a sub-micron tapered fiber, which is fabricated using

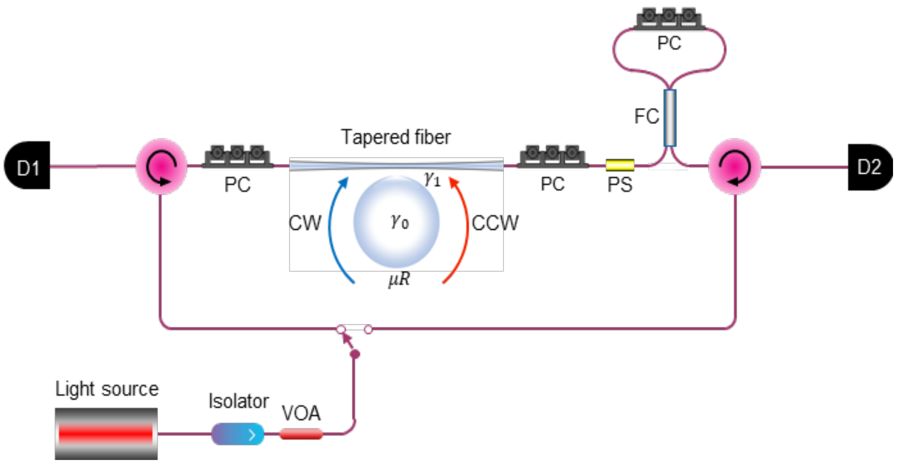

Figure S1 | *Experimental setup used in this study to investigate chiral perfect absorption on an exceptional surface (ES).* μR *: microresonator; CW: clockwise; CCW: counterclockwise; VOA: variable optical attenuator; PC: polarization controller; PS: phase shifter;* $D1$ *&* $D2$ *; photodetectors; and FC: 2-to-2 fiber coupler. See the text for details.*

heat-and-pull method. The detector D_1 is used to monitor reflection (transmission) spectra whereas the detector D_2 is used to monitor the transmission (reflection) spectra for light fields input in the CW (CCW) direction (i.e., left incidence: CW and right incidence: CCW). The fiber-loop with a polarization controller simulates a symmetric tuneable end-mirror. Reflectivity of the end-mirror is the same for light input in the CW and CCW directions. Similarly, the transmittivity for CW and CCW inputs is the same. The reflection magnitude and phase of this end-mirror are tuned using a polarization controller and a phase shifter (PS). In this way, the coupling from the CW input to the CCW direction is controlled by tuning the reflectivity of the end-mirror in the range 0 to 1.0. Polarization controllers (PC) are used at each end of the tapered fiber to set the proper polarization and to correct if any polarization rotation takes place. VOA placed after the laser is used to control the input power. The isolator in front of the laser diode is placed to prevent or minimize reflections from the optical components into the laser. Optical switch placed after the VOA is used to select the input direction (i.e., CW or CCW). We note this platform allows us to independently control and tune the waveguide-resonator coupling and the strength of unidirectional coupling between the CW and CCW modes of the resonator.

b) *Theoretical Model and numerical simulations*

Our system can be described with the coupled differential equations,

$$\frac{da_{cw}}{dt} = -(i\Delta + \Gamma)a_{cw} - \sqrt{\gamma_1}a_{in,cw} \tag{S1}$$

$$\frac{da_{ccw}}{dt} = -(i\Delta + \Gamma)a_{ccw} - \sqrt{\gamma_1}a_{in,ccw} \tag{S2}$$

where a_{cw} and a_{ccw} are the field amplitudes of the clockwise (CW) and counter-clockwise (CCW) modes in the resonator; $a_{in,cw}$ and $a_{in,ccw}$ are the field amplitudes of the inputs to the resonator in the CW (forward) and CCW (backward) directions; Δ is the detuning between the laser and resonance frequency; and $\Gamma = (\gamma_0 + \gamma_1)/2$ with γ_1 denoting the resonator-waveguide coupling loss and γ_0 denoting all other losses except the coupling.

Using the input-output formalism, we have $a_{out,cw} = a_{in,cw} + \sqrt{\gamma_1}a_{cw}$ and $a_{out,ccw} = d_{ccw} = a_{in,ccw} + \sqrt{\gamma_1}a_{ccw}$ as the outputs for the CW and CCW input directions, respectively, with d_{ccw} representing the field detected at the photodetector D_1. We note that $a_{out,ccw} = d_{ccw}$ represents the transmission for the input in the backward direction (CCW input) and reflection for the input in the forward direction (CW input). Thus D_1 measures the transmission (reflection) spectrum for the CCW (CW) input. A fiber-loop reflector with splitting coefficients r and t satisfying $|r|^2 + |t|^2 = 1$ splits $a_{out,cw}$ into two paths: The field in the first path, which is given by $d_{cw} = te^{i\phi}a_{out,cw}$ goes directly to the photodetector D_2 and represents the transmitted field in the forward direction. The field $re^{i\phi}a_{out,cw}$ in the second path propagates through a tuneable phase shifter (PS) with phase ϕ forming the back-reflected field $a_{r,cw} = re^{i2\phi}a_{out,cw}$ in the CCW direction for an input in the forward direction (CW input). For the input $a_{in,ccw}$ in the CCW direction (backward input), the field input to the tapered fiber waveguide is then given by $te^{i\phi}a_{in,ccw}$ and the reflected field is given as $ra_{in,ccw}$.

i. ***<u>Input in the CW direction (left incidence, forward direction)</u>***

We first consider the case of input only in the forward direction (CW input). A portion of the $a_{out,cw}$ will be reflected into the CCW direction through the fiber-loop reflector as $a_{r,cw} =$

$re^{i2\phi}a_{out,cw}$. Therefore, when considering the system, we need to modify the rate equation describing the CCW field in the resonator as:

$$\frac{da_{cw}}{dt} = -(i\Delta + \Gamma)a_{cw} - \sqrt{\gamma_1}a_{in,cw} \tag{S3}$$

$$\frac{da_{ccw}}{dt} = -(i\Delta + \Gamma)a_{ccw} - \sqrt{\gamma_1}a_{r,cw} = -(i\Delta + \Gamma)a_{ccw} - \sqrt{\gamma_1}re^{i2\phi}a_{out,cw} \tag{S4}$$

At steady state ($da_{cw}/dt = 0$ and $da_{ccw}/dt = 0$) we find a_{cw} and a_{ccw} as

$$a_{cw} = -\frac{\sqrt{\gamma_1}}{i\Delta+\Gamma}a_{in,cw} \tag{S5}$$

$$a_{ccw} = -\frac{\sqrt{\gamma_1}}{i\Delta+\Gamma}a_{r,cw} = -\frac{\sqrt{\gamma_1}}{i\Delta+\Gamma}re^{i2\phi}a_{out,cw} = -\frac{\sqrt{\gamma_1}}{i\Delta+\Gamma}re^{i2\phi}\left(a_{in,cw} + \sqrt{\gamma_1}a_{cw}\right)$$

$$= -re^{i2\phi}\frac{\sqrt{\gamma_1}}{i\Delta+\Gamma}\left(1 - \frac{\gamma_1}{i\Delta+\Gamma}\right)a_{in,cw} \tag{S6}$$

Similarly, output in the CCW direction (corresponding to back-reflected light for the CW input) is expressed in the modified input-output relation as

$$a_{out,ccw} = d_{ccw} = a_{r,cw} + \sqrt{\gamma_1}a_{ccw} = \left(1 - \frac{\gamma_1}{i\Delta+\Gamma}\right)a_{r,cw} = \left(1 - \frac{\gamma_1}{i\Delta+\Gamma}\right)re^{i2\phi}a_{out,cw} \tag{S7}$$

$$= \left(1 - \frac{\gamma_1}{i\Delta+\Gamma}\right)re^{i2\phi}\left(a_{in,cw} + \sqrt{\gamma_1}a_{cw}\right) = \left(1 - \frac{\gamma_1}{i\Delta+\Gamma}\right)re^{i2\phi}\left[a_{in,cw} - \frac{\gamma_1}{i\Delta+\Gamma}a_{in,cw}\right] \tag{S8}$$

$$= re^{i2\phi}\left(1 - \frac{\gamma_1}{i\Delta+\Gamma}\right)^2 a_{in,cw} \tag{S9}$$

Thus, the field at D_1 for the input in CW direction is $a_{out,ccw} = re^{i2\phi}\left(1 - \frac{\gamma_1}{i\Delta+\Gamma}\right)^2 a_{in,cw}$ from which we find the reflection spectrum R_{cw} of the system for the CW input as:

$$R_{cw} = \left|\frac{d_{ccw}}{a_{in,cw}}\right|^2 = \left|\frac{a_{out,ccw}}{a_{in,cw}}\right|^2 = |r|^2 \left|\left(1 - \frac{\gamma_1}{i\Delta+\Gamma}\right)^2\right|^2 = |r|^2 \left|1 - \frac{2\gamma_1}{i\Delta+\Gamma} + \frac{{\gamma_1}^2}{(i\Delta+\Gamma)^2}\right|^2 \quad \text{(S10)}$$

$$= |r|^2 \left[\frac{\Delta^2+(\Gamma-\gamma_1)^2}{\Gamma^2+\Delta^2}\right]^2 = |r|^2 \left[\frac{4\Delta^2+(\gamma_0-\gamma_1)^2}{4\Delta^2+(\gamma_0+\gamma_1)^2}\right]^2 \quad \text{(S11)}$$

Similarly, we can describe the field at D_2 as

$$d_{cw} = e^{i\phi} t a_{out,cw} = e^{i\phi} t\left(a_{in,cw} + \sqrt{\gamma_1} a_{cw}\right) = e^{i\phi} t \left(1 - \frac{\gamma_1}{i\Delta+\Gamma}\right) a_{in,cw} \quad \text{(S12)}$$

Then the transmission spectrum T_{cw} of the system is found as

$$T_{cw} = \left|\frac{d_{cw}}{a_{in,cw}}\right|^2 = |t|^2 \left|1 - \frac{\gamma_1}{i\Delta+\Gamma}\right|^2 = |t|^2 \left[\frac{4\Delta^2+(\gamma_0-\gamma_1)^2}{4\Delta^2+(\gamma_0+\gamma_1)^2}\right] \quad \text{(S13)}$$

Note the extra term of $\frac{{\gamma_1}^2}{(i\Delta+\Gamma)^2}$ in the expression for R_{cw} compared to T_{cw} already implies that the lineshape of R_{cw} will be significantly different than that of T_{cw}. Using the relation $A_{cw} + T_{cw} + R_{cw} = 1$, we can write the absorption for the input in the CW direction as:

$$A_{cw} = 1 - T_{cw} - R_{cw} = 1 - |t|^2 \left[\frac{4\Delta^2+(\gamma_0-\gamma_1)^2}{4\Delta^2+(\gamma_0+\gamma_1)^2}\right] - |r|^2 \left[\frac{4\Delta^2+(\gamma_0-\gamma_1)^2}{4\Delta^2+(\gamma_0+\gamma_1)^2}\right]^2 \quad \text{(S14)}$$

which reveals that the absorption spectrum is a superposition of a Lorentzian (i.e., T_{cw} spectrum) and a squared Lorentzian function (i.e., R_{cw} spectrum). This implies that one can set the lineshape of the absorption spectrum by finely tuning the system parameters. For example, when the system is set at critical coupling ($\gamma_0 = \gamma_1$, that is $\Gamma = \gamma_0 = \gamma_1$), absorption A_{cw} is written as

$$A_{cw(\gamma_0=\gamma_1)} = 1 - |t|^2 \left(\frac{1}{1+\gamma_0^2/\Delta^2}\right) - |r|^2 \left(\frac{1}{1+\gamma_0^2/\Delta^2}\right)^2 \quad \text{(S15)}$$

which with the choice of completely reflecting end-mirror ($t = 0, r = 1$) reduces to $A_{cw(\gamma_0=\gamma_1)} = 1 - \left(\frac{1}{1+\gamma_0^2/\Delta^2}\right)^2$, implying a squared-Lorentzian lineshape, and with the choice of completely transmitting end-mirror ($t = 1$, $r = 0$) reduces to $A_{cw(\gamma_0=\gamma_1)} = 1 - \left(\frac{1}{1+\gamma_0^2/\Delta^2}\right)$, implying a Lorentzian lineshape. Thus, provided that $\gamma_0/\gamma_1$is kept constant, one can tune the absorption lineshape from a Lorentzian to a squared-Lorentzian form by varying reflectivity of the end-mirror (**Fig.S2**). Similarly, one can tune the lineshape by tuning the waveguide-resonator coupling strength (varying γ_0/γ_1) if r and t are kept constant (**Fig. S3**).

ii. *<u>Input in the CCW direction (left incidence, backward direction)</u>*

We now consider the case that we have input only in the backward direction (CCW input). The output field $a_{out,ccw}$ goes directly

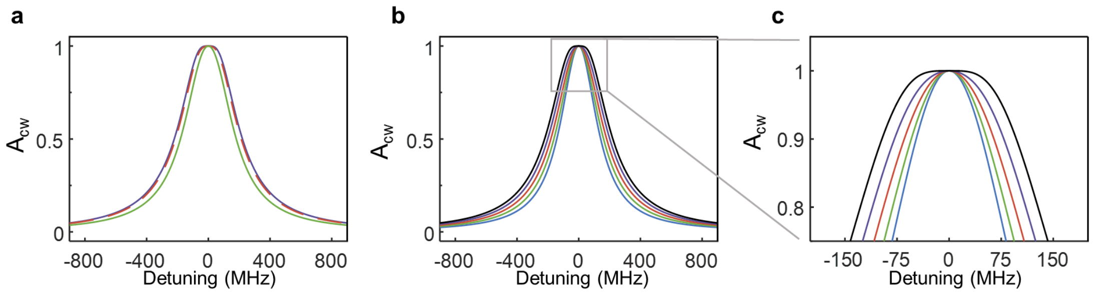

***Figure S2 |** Effect of the reflectivity of the end-mirror on the normalized absorption spectra obtained on the exceptional surface (ES) at critical coupling for CW input. **a**, Calculated absorption spectra simulating experimentally investigated end-mirror: Fully-reflecting end-mirror ($|r|^2 = 1, |t|^2 = 0$) (purple), 50:50 half-mirror ($|r|^2 = |t|^2 = 1/2$) (green), and 10:90 mirror ($|r|^2 = 0.9, |t|^2 = 0.1$) (dashed red). **b**, Calculated absorption spectra simulating an end-mirror with different reflectivity values, corresponding to various settings of the fiber-loop reflector: $|r|^2$ equals to 0 (blue), $1/4$ (green), $1/2$ (red), $3/4$ (purple), and 1 (black). These settings simulate end-mirrors with zero-reflection (blue), 25% reflection (green), 50% reflection (red), 75% reflection (purple), and 100% reflection (black), respectively. **c**, Enlarged view of the top part of the spectra shown in **b**. Transition of the spectra from Lorentzian lineshape to a flat-top quartic lineshape is clearly seen as the reflectivity of the end-mirror is increased.*

to the detector D_1(i.e., no back-reflection into the CW mode). The field input to the waveguide after the fiber-loop reflector is $te^{i\phi}a_{in,ccw}$. There is no input in the CW direction, thus we have $a_{in,cw} = 0$ and $a_{cw} = 0$ (i.e., no coupling between the CW and CCW modes and input only in the CCW direction). Thus, it is enough to consider only the modified rate equation:

$$\frac{da_{ccw}}{dt} = -(i\Delta + \Gamma)a_{ccw} - \sqrt{\gamma_1}te^{i\phi}a_{in,ccw} \tag{S16}$$

At steady state ($da_{ccw}/dt = 0$) we find a_{cw} and a_{ccw} as

$$a_{ccw} = -\frac{\sqrt{\gamma_1}te^{i\phi}}{(i\Delta+\Gamma)}a_{in,ccw} \tag{S17}$$

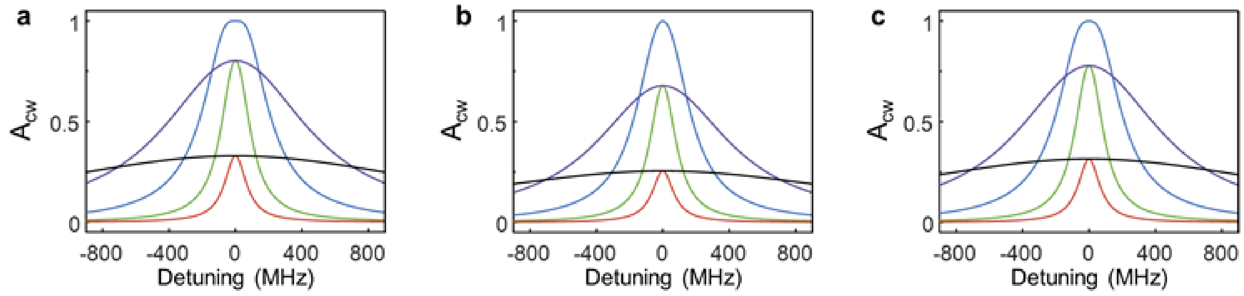

***Figure S3** | Effect of the waveguide-resonator coupling regime on the normalized absorption spectra. Coupling regime is quantified by the ratio γ_0/γ_1 (ratio of the resonator losses γ_0 including material, scattering and radiation, to the waveguide-resonator coupling loss γ_1). In the simulation results shown in **a**, **b**, and **c**, we used $\gamma_0/\gamma_1 = 1$ (critical coupling, blue), $\gamma_0/\gamma_1 = 5$ (undercoupling, green), $\gamma_0/\gamma_1 = 20$ (deep undercoupling, red), $\gamma_0/\gamma_1 = 1/5$ (overcoupling, purple), and $\gamma_0/\gamma_1 = 1/20$ (deep overcoupling, black). Calculated absorption spectra simulating experimentally investigated end-mirrors: **a**, Fully reflecting end-mirror ($|r|^2 = 1, |t|^2 = 0$), **b**, 50:50 half-mirror ($|r|^2 = |t|^2 = 1/2$), and **c**, 10:90 end-mirror with 90% reflection ($|r|^2 = 0.9, |t|^2 = 0.1$). Flat-top quartic lineshape is clearly seen at the critical coupling $\gamma_0/\gamma_1 = 1$ in **a** and **c** but not in **b**, implying the effect of end-mirror reflectivity in the process. As the system is moved to undercoupling or overcoupling regime, the flat-top becomes obscured and the lineshape becomes Lorentzian.*

Substituting into the modified input-output relation $a_{out,ccw} = d_{ccw} = te^{i\phi} a_{in,ccw} + \sqrt{\gamma_1} a_{ccw}$:

$$a_{out,ccw} = te^{i\phi} a_{in,ccw} + \sqrt{\gamma_1} a_{ccw} = te^{i\phi} a_{in,ccw} - \frac{\gamma_1 te^{i\phi}}{(i\Delta+\Gamma)} a_{in,ccw} \tag{S18}$$

$$= te^{i\phi} \left(1 - \frac{\gamma_1}{(i\Delta+\Gamma)}\right) a_{in,ccw} \tag{S19}$$

The field detected at D_1 for the input in CCW direction gives the transmission spectrum T_{ccw} as:

$$T_{ccw} = \left|\frac{d_{ccw}}{a_{in,cw}}\right|^2 = \left|\frac{a_{out,ccw}}{a_{in,cw}}\right|^2 = |t|^2 \left|\left(1 - \frac{\gamma_1}{(i\Delta+\Gamma)}\right)\right|^2 = |t|^2 \frac{\Delta^2+(\Gamma-\gamma_1)^2}{\Gamma^2+\Delta^2} \tag{S20}$$

$$= |t|^2 \left(\frac{4\Delta^2+(\gamma_0-\gamma_1)^2}{4\Delta^2+(\gamma_0+\gamma_1)^2}\right) \tag{S21}$$

For the CCW input, we calculate the reflection R_{ccw} as

$$R_{ccw} = \left|\frac{re^{i\phi} a_{in,ccw}}{a_{in,ccw}}\right|^2 = |r|^2 \tag{S22}$$

which is constant for all frequencies and is significantly different from R_{cw} which exhibits a squared Lorentzian lineshape. We can then use the relation $A_{ccw} + T_{ccw} + R_{ccw} = 1$ to write absorption for the input in the CCW direction as

$$A_{ccw} = 1 - T_{ccw} - R_{ccw} = 1 - |r|^2 - |t|^2 \left(\frac{4\Delta^2+(\gamma_0-\gamma_1)^2}{4\Delta^2+(\gamma_0+\gamma_1)^2}\right) = |t|^2 \left(1 - \frac{4\Delta^2+(\gamma_0-\gamma_1)^2}{4\Delta^2+(\gamma_0+\gamma_1)^2}\right) \tag{S23}$$

which reveals an absorption spectrum with Lorentzian lineshape. At critical coupling ($\gamma_0 = \gamma_1$, that is $\Gamma = \gamma_0 = \gamma_1$), absorption spectra A_{ccw} for a CCW input becomes

$$A_{ccw(\gamma_0=\gamma_1)} = |t|^2 \left(1 - \frac{\Delta^2}{\Delta^2+\gamma_0^2}\right) = |t|^2 \left(\frac{\gamma_0^2}{\Delta^2+\gamma_0^2}\right) = |t|^2 \left(\frac{1}{1+\Delta^2/\gamma_0^2}\right) \tag{S24}$$

iii. ***Intracavity field intensity at exceptional surfaces of different coupling regimes***

We performed numerical simulations of the system using COMSOL Multiphysics which allowed

us to observe the intracavity field for CW and CCW inputs at different coupling regimes and hence on different exceptional surfaces (**Figs. S4 and S5**). We performed the simulations for two specific cases, that is for a perfectly reflecting end-mirror (**Fig. S4**) and for an end-mirror with half reflecting and half transmitting, $|r|^2 = |t|^2 = 1/2$ (**Fig.S5**). Simulations show the formation of a standing-wave like pattern inside the resonator only for the CW input. This is because, the reflector is placed at only one of the waveguide ends (the end in the CW direction) and thus the light transmitted through the waveguide-coupled resonator in the CW direction is back reflected in the CCW direction. As a result, there are two fields propagating in the CW and CCW directions in the resonator even if the input to the system is in the CW direction. The response of the system for these end-mirrors (i.e., the fully reflective and the partially reflective end-mirrors) differ significantly for the CCW input: In **Fig. S4** there is no field in the resonator (thus no absorption for CCW input) because the CCW input is fully reflected and does not reach the resonator. In **Fig. S5**, while half of the input

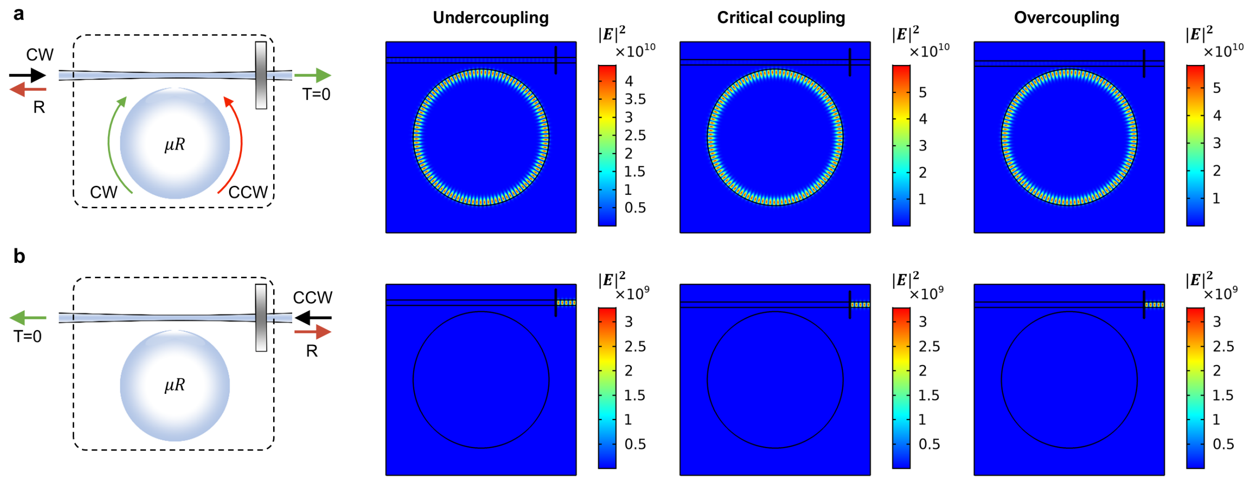

***Figure S4 |** Intracavity field intensity for **a**, clockwise CW and **b**, counterclockwise CCW input on the exceptional surfaces associated with different waveguide-microresonator (μR) coupling regimes for a system with fully reflective end-mirror. Dotted boxes in the left panels represent the ES-device composed of a waveguide-coupled resonator with the end-mirror. Black arrows denote the CW and CCW input ports of the ES-device. Intracavity field intensity for the CW input is highest at the critical coupling. Note the absence of field inside the resonator for the CCW input.*

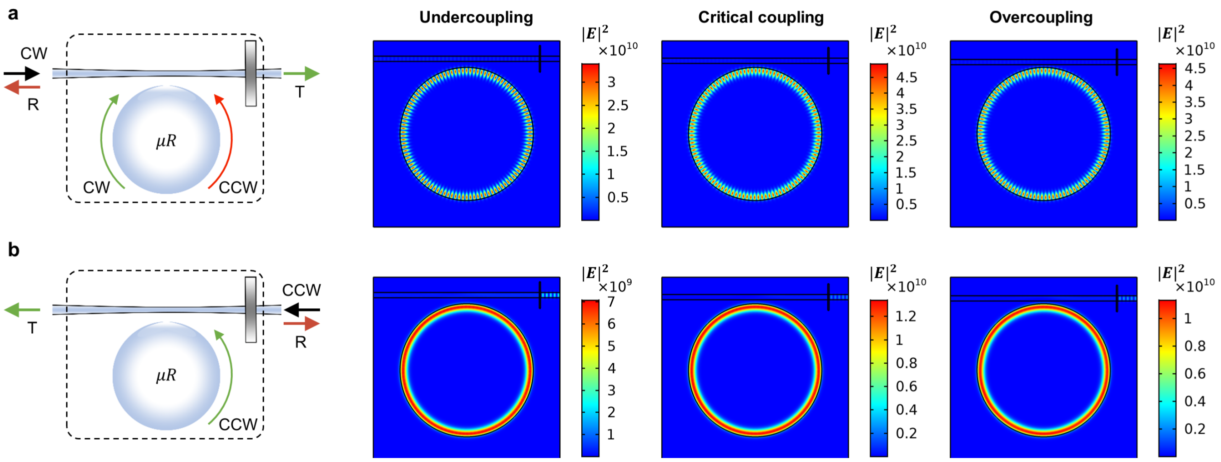

Figure S5 | *Intracavity field intensity for* ***a****, clockwise CW and* ***b****, counterclockwise CCW input on the exceptional surfaces associated with different waveguide-microresonator (μR) coupling regimes when the end-miror is half reflecting and half transmitting. Dotted boxes in the left panels represent the ES-device composed of a waveguide-coupled resonator with the end-mirror. Black arrows denote the CW and CCW input ports of the ES-device. Intensity for the CW input is highest at critical coupling. Note the standing-wave like pattern for the CW input and the traveling wave pattern for the CCW input.*

CCW field is reflected back to the input port, the other half travels and couples into the resonator in the CCW direction (thus there is absorption for CCW input). Since there is no end-mirror in the CCW input direction, there is only CCW traveling wave in the resonator for the CCW input (**Fig. S5**). This asymmetric response for the CCW input is observed at all exceptional surfaces associated with waveguide-resonator coupling regimes. Simulations also show that the ES emerging at the critical coupling leads to the highest intracavity-field intensity for CW input. On a given ES associated with a coupling strength, the intracavity field intensity is stronger for the CW input than for the CCW input.

c) Normalization procedure to assess absorption in experimentally obtained spectra

To correctly assess the absorption on exceptional surfaces, it is important that all losses in the system (i.e., including the insertion and return losses of various components and the propagation

losses in the connecting fibers but not the resonator-related losses) incurred during light propagation in the experimental setup are measured and are considered in the normalization process. We have measured these spurious losses in the path of the fields for input both in the CW and CCW directions from the setup input-point until the detection at the detectors D_1 and D_2. Since these paths are different for different input directions, the losses are different, and they should be measured individually and included in the normalization of the associated experimentally obtained spectra. We measured these losses by recording off-resonant (i.e., without the resonator) transmission (reflection) and reflection (transmission) for the input in the CW (CCW) direction at detectors D_1 and D_2, respectively.

i. *Input in the Clockwise (CW) Direction*

If there were no losses at all in the system, off-resonant transmitted and reflected field intensities for an input with intensity $I_{in/cw}$ in the CW direction would be given as $I_{T/cw-off-\,lossless} = |t|^2 I_{in/cw}$ and $I_{R/cw-off-\,lossless} = |r|^2 I_{in/cw}$. Due to the presence of spurious losses (i.e., all those losses in the setup except for those induce by the presence of the resonator) the off-resonant transmission and reflection field intensities are reduced to the values $I_{T/cw-off}$ and $I_{R/cw-off}$, which we measure by removing the resonator from the setup. These non-resonant spurious losses are then taken into account by multiplying the transmitted field intensity $I_{T/cw}(\omega)$ and the reflected field intensity $I_{R/cw}(\omega)$, both measured in the presence of the resonator, with $\left(I_{T/cw-off-\,lossless}/I_{T/cw-off}\right)$ and $\left(I_{R/cw-off-\,lossless}/I_{R/cw-off}\right)$. Thus, transmission and reflection spectra normalized with respect to the input $I_{in/cw}$ after taking the spurious losses into account are given by the normalized transmission $T_{cw}(\omega)$

$$T_{cw}(\omega) = \frac{I_{T/cw}(\omega)\left(\frac{I_{T/cw-off-lossless}}{I_{T/cw-off}}\right)}{I_{in/cw}} = \frac{I_{T/cw}(\omega)\left(\frac{|t|^2 I_{in/cw}}{I_{T/cw-off}}\right)}{I_{in/cw}} = \frac{I_{T/cw}(\omega)}{I_{T/cw-off}}|t|^2 \tag{25}$$

and the normalized reflection $R_{cw,N}(\omega)$

$$R_{cw}(\omega) = \frac{I_{R/cw}(\omega)\left(\frac{I_{R/cw-off-lossless}}{I_{R/cw-off}}\right)}{I_{in/cw}} = \frac{I_{R/cw}(\omega)\left(\frac{|r|^2 I_{in/cw}}{I_{R/cw-off}}\right)}{I_{in/cw}} = \frac{I_{R/cw}(\omega)}{I_{R/cw-off}}|r|^2 \tag{26}$$

In our experiments, effective reflection and transmission by the end-mirror is controlled by polarization controllers in the fiber loop. We can tune the reflectivity of the end-mirror that controls the coupling of the CW light into the CCW between 0 and 1. The normalized absorption spectrum is then given as $A_{cw}(\omega) = 1 - T_{cw}(\omega) - R_{cw}(\omega)$:

$$A_{cw}(\omega) = 1 - \frac{I_{T/cw}(\omega)}{I_{T/cw-off}}|t|^2 - \frac{I_{R/cw}(\omega)}{I_{R/cw-off}}|r|^2 = 1 - \frac{I_{T/cw}(\omega)}{I_{T/cw-off}} + \left(\frac{I_{T/cw}(\omega)}{I_{T/cw-off}} - \frac{I_{R/cw}(\omega)}{I_{R/cw-off}}\right)|r|^2 \quad (27)$$

.

ii. *Input in the Counterclockwise (CCW) Direction*

Since the paths for the CW and CCW input fields are different, the losses they experience are different, too. For CCW input, off-resonant transmission measured at D_1 helps determine the spurious losses for the CCW input in the system before the resonator is inserted. This loss is given as $I_{T/ccw-off-lossless} = |t|^2 I_{in/ccw}$. As for CW input, we take these spurious losses into account by multiplying the transmitted field intensity $I_{T/ccw}(\omega)$ and the reflected field intensity $I_{R/ccw}(\omega)$, both measured in the presence of the resonator, with $\left(I_{T/ccw-off-\,lossless}/I_{T/ccw-off}\right)$ and $\left(I_{R/ccw-off-\,lossless}/I_{R/ccw-off}\right)$. Here, $I_{T/ccw-off}$ and $I_{R/ccw-off}$ denote the off-resonant transmitted and reflected field intensities, respectively. We note that in the case of CCW input, the resonator does not play a role and $I_{R/ccw} = I_{R/ccw-off}$. Following the procedure outlined for CW input above, we find the expression for the normalized transmission spectra for the input in CCW direction as

$$T_{ccw}(\omega) = \frac{I_{T/ccw}(\omega)\left(\frac{I_{T/ccw-off-lossless}}{I_{T/ccw-off}}\right)}{I_{in/ccw}} = \frac{I_{T/ccw}(\omega)\left(\frac{|t|^2 I_{in/ccw}}{I_{T/ccw-off}}\right)}{I_{in/ccw}} = \frac{I_{T/ccw}(\omega)}{I_{T/ccw-off}}|t|^2 \quad (28)$$

The normalized reflection spectrum is just given as

$$R_{ccw}(\omega) = \frac{I_{R/ccw}(\omega)\left(\frac{I_{R/ccw-off-lossless}}{I_{R/ccw-off}}\right)}{I_{in/ccw}} = \frac{I_{R/ccw}(\omega)\left(\frac{|r|^2 I_{in/ccw}}{I_{R/ccw-off}}\right)}{I_{in/ccw}} = \frac{I_{R/ccw}(\omega)}{I_{R/ccw-off}}|r|^2 = |r|^2 \quad (29)$$

where we used the fact that for the CCW input $I_{R/ccw}(\omega) = I_{R/ccw-off}$ because resonator is not involved. Then we calculate the normalized absorption spectrum as

$$A_{ccw}(\omega) = 1 - T_{ccw}(\omega) - R_{ccw}(\omega) = 1 - \frac{I_{T/ccw}(\omega)}{I_{T/ccw-off}}|t|^2 - |r|^2 = \left(1 - \frac{I_{T/ccw}(\omega)}{I_{T/ccw-off}}\right)|t|^2 \quad (30)$$

II. Supplementary experimental data

Here we provide supplementary experimental data and evidence that support the conclusions of the study as outlined in the main text.

a) *Effect of end-mirror reflectivity on the reflection spectra obtained on the ES associated with critical coupling for CW input*

The theoretical model predicts that when the system is at critical coupling, reflection spectra on the ES exhibits squared Lorentzian lineshape with perfect absorption occurring at the ES frequency. The experimentally obtained reflection spectra R_{cw} reveals the expected squared Lorentzian lineshape (i.e., flat bottom) at the critical coupling at all end-mirror reflectivity values (**Fig. S6**). As seen in the normalized reflection spectra, we have $R_{cw} = 0$ at the ES-frequency. Moreover, T_{cw} at the ES-frequency is zero because the system is at critical coupling. Then, using $A_{cw} = 1 - R_{cw} - T_{cw}$, we find that perfect absorption occurs at the ES-frequency for the CW input. Reflection and thus absorption at other frequencies depends on the end-mirror reflectivity which is controlled in our experiments by the fiber-loop reflector.

b) *Effect Exceptional surfaces obtained from reflection spectra at different resonator-waveguide coupling regimes*

As we change the coupling strength between the resonator and the waveguide, we effectively modify the imaginary part of the diagonal elements of the effective Hamiltonian of the system given in the main text as

$$H_{ES} = \begin{pmatrix} \omega_0 - i\Gamma & 0 \\ \kappa & \omega_0 - i\Gamma \end{pmatrix} \quad (31)$$

where $\Gamma = (\gamma_0 + \gamma_1)/2$ is modified as the coupling regime is changed by varying the resonator-waveguide gap (i.e., varying γ_1). We have obtained exceptional surfaces at the critical coupling

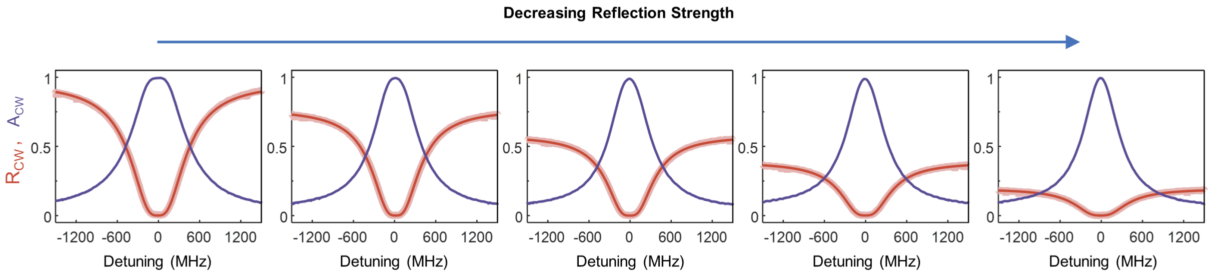

Figure S6 | Experimentally obtained normalized reflection spectra R_{cw} and the calculated absorption spectra A_{cw} on the exceptional surface associated with critical coupling at various values of the reflection magnitude $|r|$ of the end-mirror. Reflection spectra exhibit squared Lorentzian lineshapes with flat bottom at all non-zero $|r|$. As $|r|$ decreases the squared Lorentzian features of absorption spectra become vague. Perfect absorption takes place at the ES-frequency at all non-zero values of $|r|$. Reflection and absorption at frequencies other than the ES-frequency depend on $|r|$.

(**Fig. 2** in the main text), as well as in the undercoupling (**Fig. S7**) and overcoupling (**Fig. S8**) regimes. We note that at each resonator-waveguide coupling strength, a new ES emerges. These exceptional surfaces differ in the imaginary parts of their complex ES eigenfrequencies. In the experiments, we reconstructed ES in the 2D parameter $\{|r|,\phi\}$ space of the system from the normalized reflection spectra R_{cw} for the CW input (input in the forward direction, left incidence). $|r|$ and ϕ are controlled by fiber-loop reflector and a PS (**Fig. S1**). Curve fitting to the experimentally obtained R_{cw} is used to estimate the real and imaginary parts of the complex eigenfrequency of the system on the ES where two eigenfrequencies coalesce (see the Main text for details). For all studied coupling regimes, we find that Δw (i.e., difference of real parts) values are in the range $[-7.4MHz, 6.9MHz]$ and $\Delta\Gamma$ (i.e., difference of imaginary parts) values are in the range $[-5.5MHz, 8.8MHz]$, which, when normalized with the frequency ω_0 and linewidth Γ of the resonance without the end-mirror, yield $|\Delta w/\omega_0| \lesssim 10^{-8}$ and $\Delta\Gamma/\Gamma \lesssim 10^{-2}$. This reveals that our system is indeed on the ES.

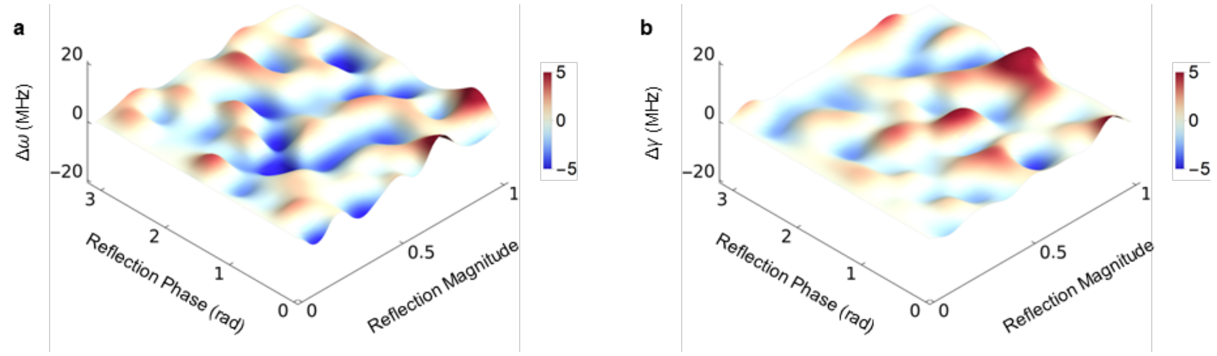

Figure S7* | *Experimentally obtained exceptional surface from the reflection spectra in the undercoupling regime. ***a****, Δw and* ***b****, Δγ correspond to the difference between the real and imaginary parts of two complex eigenfrequencies obtained from curve fitting to R_{cw} measured at various reflection phases ϕ and magnitudes $|r|$ of the end-mirror.*

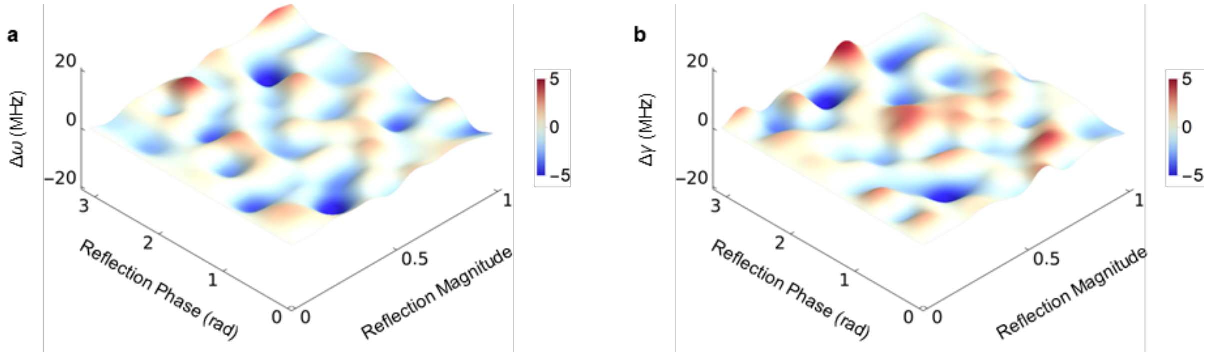

Figure S8* | *Experimentally obtained exceptional surface from the reflection spectra in the overcoupling regime. ***a****, Δw and* ***b****, Δγ correspond to the difference between the real and imaginary parts of two complex eigenfrequencies obtained from curve fitting to R_{cw} measured at various reflection phases ϕ and magnitudes $|r|$ of the end-mirror.*

c) *Reflection and absorption spectra at different resonator-waveguide coupling regimes with a fully reflecting end-mirror*

If all parameters of the system are kept constant but only the resonator-waveguide coupling strength is modified, a new ES will emerge at each coupling regime. However, only the ES at the

critical coupling will lead to perfect absorption with quartic (i.e., squared Lorentzian) lineshape (**Fig. S9**). We experimentally obtained the reflection spectra R_{cw} and calculated the absorption spectra A_{cw} as $A_{cw} = 1 - R_{cw}$ (Note that in this case $T_{cw} = 0$). As the system moves away from the critical coupling, the ES emerging at the new coupling regimes does not satisfy the perfect absorption condition. We also observe that while the ES at the critical coupling clearly exhibits a quartic lineshape in the reflection and absorption spectra, this feature is not clear as the system moves away from critical coupling.

d) *Reflection, transmission, and absorption spectra on ES at different resonator-waveguide coupling regimes with a 50:50 end-mirror*

In the main text, **Figs. 2a** & **3** were obtained for fully reflective end-mirror whereas **Fig. 4** was obtained with a 10:90 end-mirror with 10% transmission and 90% reflection for both the CCW and CW input directions. Here, in **Fig. S10,** we provide the experimental results for a 50:50 end-mirror which reflects 50% and transmits 50% of the field input in the CW and CCW directions.

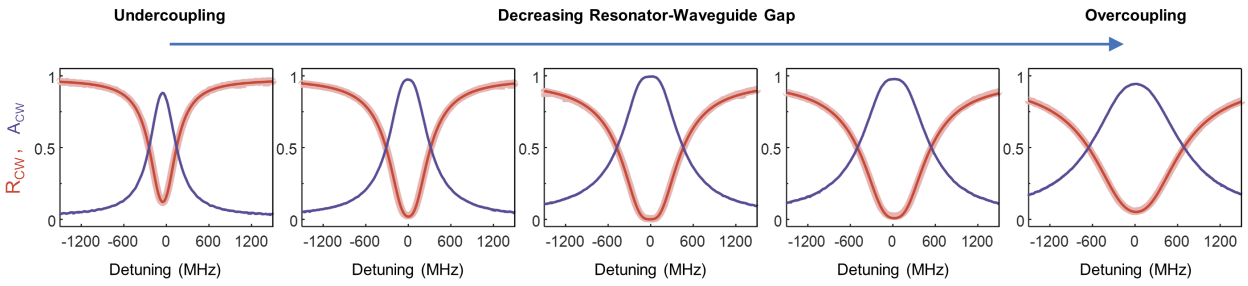

Figure S9 | *Experimentally obtained reflection and absorption spectra on exceptional surfaces associated with different resonator-waveguide coupling regimes. Quartic lineshape (squared Lorentzian) with flat bottom resonance dip for the reflection spectra R_{cw} (red) and with flat top resonance peak for absorption spectra A_{cw} (purple) is clearly seen at the critical coupling. As the resonator-waveguide coupling strength moves away from critical coupling towards undercoupling (increasing gap) or overcoupling (decreasing gap) regimes, quartic features fade away and spectra look more like Lorentzian. CPA takes place only at critical coupling.*

Similar to the results in the main text, we observe: i) perfect absorption on the exceptional surface with quartic lineshape at the critical coupling for the CW input; ii) chiral absorption in the sense that absorption for CW input is much larger than that for CCW input on all exceptional surfaces created at different waveguide-resonator coupling; iii) while the quartic feature is dominant and is clearly seen for the reflection for the ES at the critical coupling, it becomes vague

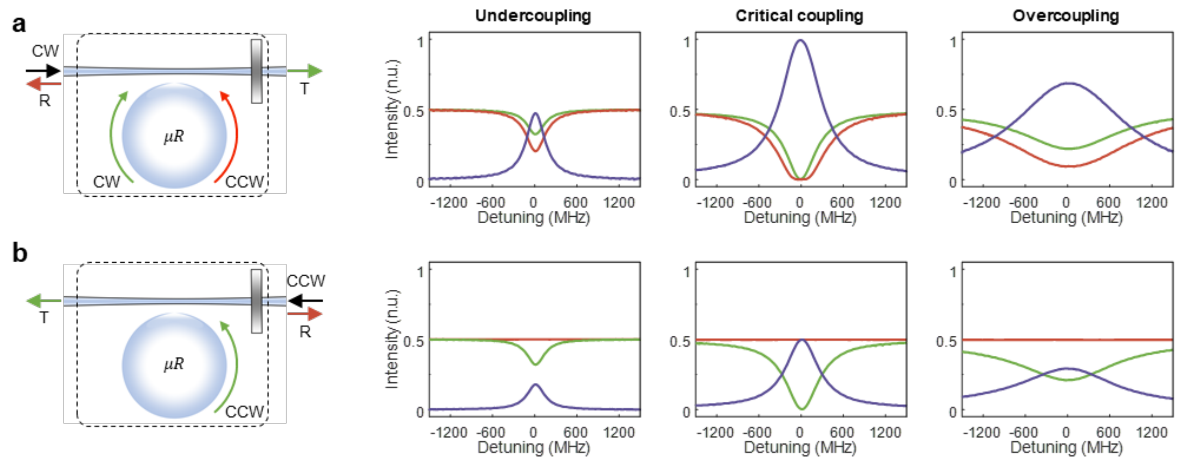

Figure S10 | *Chiral perfect absorption on exceptional surfaces at different resonator-waveguide coupling regimes for a 50:50 end-mirror. Dotted boxes in the left panels in* ***a*** *and* ***b*** *represent the ES-device composed of a waveguide-coupled microresonator (μR) with an end-mirror with 50% reflection and 50% transmission. Black arrows denote the CW and CCW input ports of the ES-device, and red and green arrows represent the corresponding reflection and transmission ports. In the case of CW input as in* ***a****, the field inside μR has both CW and CCW components whereas it has only CCW component for the CCW input as in* ***b****. Measured transmission $T_{cw(ccw)}$ (green) and reflection $R_{cw(ccw)}$ (red) spectra and calculated absorption $A_{cw(ccw)} = 1 - R_{cw(ccw)} - T_{cw(ccw)}$ (purple) spectra of the ES-device at the undercoupling, critical coupling and overcoupling regimes for CW (upper panel) and CCW (lower panel) inputs. T_{cw} and T_{ccw} have Lorentzian lineshapes with resonance dips at zero-detuning (ES frequency) at all coupling regimes; R_{ccw} is constant at all frequencies; and R_{cw} exhibits squared Lorentzian spectra. Perfect absorption on the ES is observed at the critical coupling for CW input only, implying chiral perfect absorption. A_{cw} is always larger than $A_{(ccw)}$, and hence chiral absorption at all coupling conditions.*

for exceptional surfaces obtained when the system moves away from critical coupling; and iv) T_{cw} and T_{ccw} have Lorentzian lineshapes with resonance dips at zero-detuning (ES frequency) at all coupling regimes; R_{ccw} is constant at all frequencies; and $\mathrm{R_{cw}}$ exhibits a squared Lorentzian spectrum.

e) *Reflection, transmission, and absorption spectra on exceptional surfaces with and without considering the spurious losses that are not related with the resonator*

The absorption spectra presented in the main text and the Supplement so far have been obtained using the normalization process introduced in Section I. This process considers the effect of spurious losses, which do not originate directly from the waveguide-coupled resonator with an end-mirror. These losses include insertion, return, and propagation losses induced by the optical components and connectors used in the setup. Such losses are measured without inserting the resonator into the setup. One may consider two different alternatives to the method introduced in Section I. One such approach may be to use $\tilde{A}_{cw(ccw)}(\omega) = 1 - \tilde{R}_{cw(ccw)}(\omega) - \tilde{T}_{cw(ccw)}(\omega)$ where $\tilde{R}_{cw(ccw)}(\omega) = I_{R/cw(ccw)}(\omega)/I_{in/cw(ccw)}$ and $\tilde{T}_{cw(ccw)}(\omega) = I_{T/cw(ccw)}(\omega)/I_{in/cw(ccw)}$ are simply the measured transmission and reflection, respectively, $\tilde{A}_{cw(ccw)}$ is the calculated absorption, which includes all losses. Note that the normalization is done here with respect to the input power only. Another approach may be to measure off-resonant losses $L_{cw(ccw)} = \tilde{A}_{cw(ccw)-off}$ and use it in the absorption calculation as $A'_{cw(ccw)} = 1 - \tilde{R}_{cw(ccw)}(\omega) - \tilde{T}_{cw(ccw)}(\omega) - \tilde{A}_{cw(ccw)-off}$, where the normalization in $\tilde{R}_{cw(ccw)}(\omega)$ and $\tilde{T}_{cw(ccw)}(\omega)$ is again done with respect to the input power.

Figures S11-S13 present a comparison of these different ways of calculating absorption for 0:100 (fully reflecting), 50:50, and 10:90 end-mirrors, respectively. For all the cases considered here, chiral absorption on the ES and squared Lorentzian absorption spectra are clearly seen. Perfect absorption with quartic lineshape at critical coupling is seen for the first two methods, that is for the normalization method introduced in Section I (labelled $A_{cw(ccw)}(\omega)$) and for the case when losses are not separately considered (labelled $\tilde{A}_{cw(ccw)}(\omega)$). When off-resonant losses are

included as in $A'_{cw(ccw)}(\omega) = 1 - \tilde{R}_{cw(ccw)}(\omega) - \tilde{T}_{cw(ccw)}(\omega) - \tilde{A}_{cw(ccw)-off}$, the amount of absorption $A'_{cw(ccw)}$ at the ES frequency is naturally limited by $1 - \tilde{A}_{cw(ccw)-off}$. While the absorption is still maximal at the ES frequency, it does not reach the value of perfect absorption $A'_{cw(ccw)} = 1$.

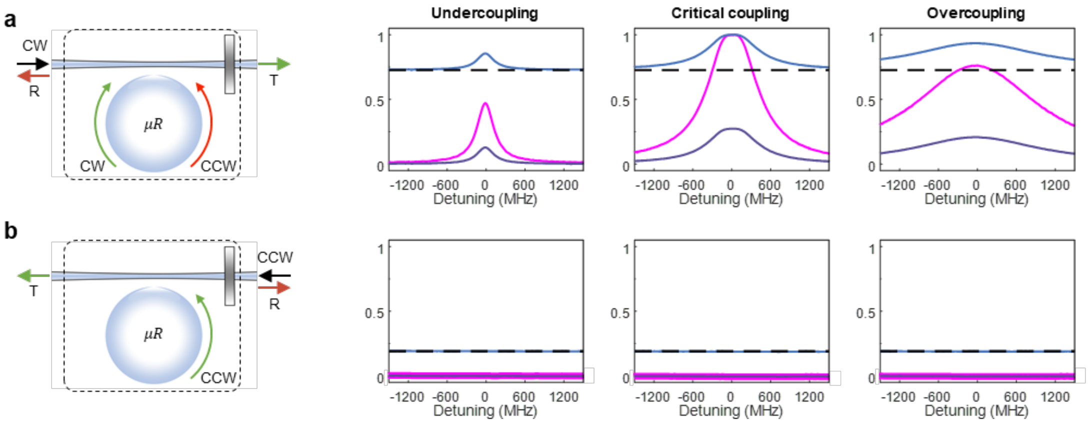

Figure S11 | *Absorption spectra calculated from experimentally obtained transmission and reflection spectra on ES for a fully reflecting end-mirror. Dashed lines denote spurious losses not directly related to the resonator. Absorption spectra are calculated using* $A_{cw(ccw)}$ *(magenta: normalization takes the losses into account. See Section I),* $\tilde{A}_{cw(ccw)}$*(blue: spectra include also spurious losses), and* $A'_{cw(ccw)}$ *(purple: spurious losses are subtracted). Absorption for the CCW input is lower than that for the CW input for all cases, and hence chiral absorption.* A_{ccw} *and* A'_{ccw} *See the text for the definitions of* $\tilde{A}_{cw(ccw)}$ *and* $A'_{cw(ccw)}$.

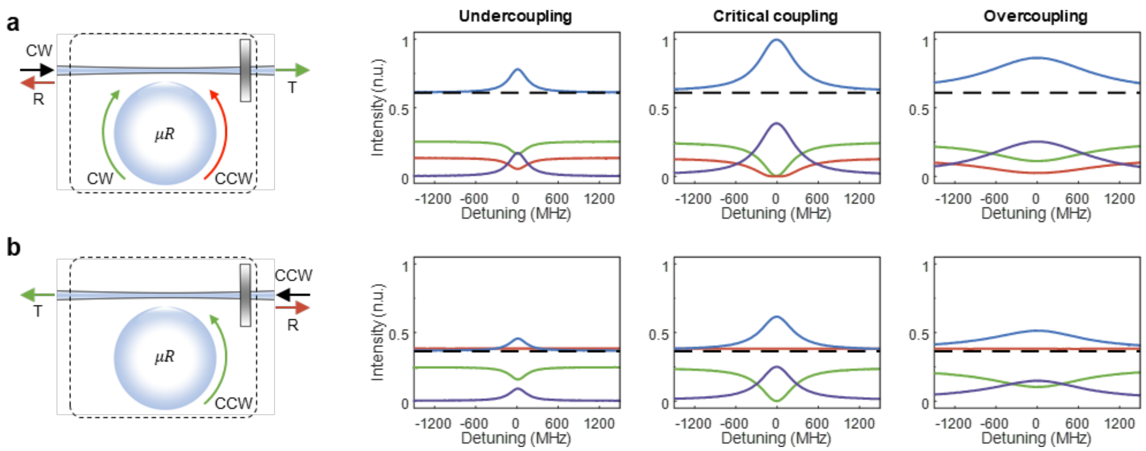

Figure S12 | *Chiral absorption on ES for a 50:50 end-mirror. Dashed lines indicate spurious losses not directly related to the resonator.* $T_{cw(ccw)}$ *(green) and* $R_{cw(ccw)}$ *(red) are measured with the resonator in place. To compare with Fig. 4 in the main text, absorption spectra are calculated here using* $\tilde{A}_{cw(ccw)}$ *(blue) or* $A'_{cw(ccw)}$ *(purple).* $\tilde{A}_{cw(ccw)}$ *reaches unity at the ES-frequency at the critical coupling. The value for* $A'_{cw(ccw)}$ *is limited by* $1-\tilde{A}_{cw(ccw)-off}$ *. For all cases the absorption for CW input is always larger than for CCW input, and hence chiral absorption.*

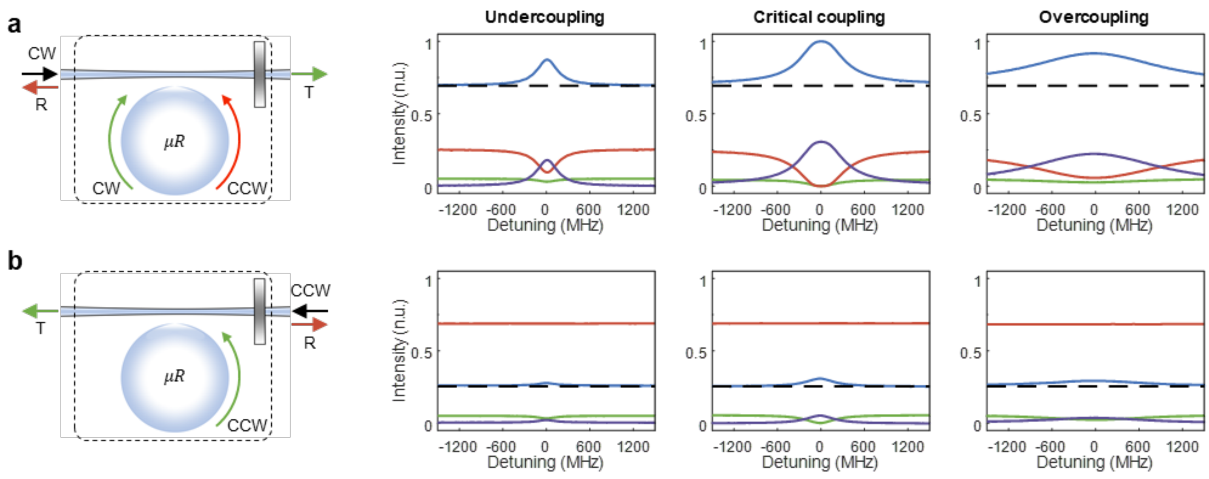

Figure S13 | *Chiral absorption on exceptional surfaces at different resonator-waveguide coupling regimes for a 10:90 end-mirror. Dashed lines denote losses not directly related to the resonator. Transmission* $T_{cw(ccw)}$ *(green) and reflection* $R_{cw(ccw)}$ *(red) spectra are measured with the resonator in place. . To compare with Fig. 4 in the main text, absorption spectra are calculated here using* $\tilde{A}_{cw(ccw)} = 1 - \tilde{R}_{cw(ccw)} - \tilde{T}_{cw(ccw)}$ *(blue) or* $A'_{cw(ccw)} = 1 - \tilde{R}_{cw(ccw)} - \tilde{T}_{cw(ccw)} - \tilde{A}_{cw(ccw)-off}$ *(purple).* $\tilde{A}_{cw(ccw)}$ *reaches unity at the ES-frequency at the critical coupling. The value for* $A'_{cw(ccw)}$ *is limited by* $1 - \tilde{A}_{cw(ccw)-off}$*. For all cases the absorption for CW input is always larger than for CCW input, and hence chiral absorption.*
